# Supplementary material for: Longitudinal microstructural changes in 18 amygdala nuclei resonate with cortical circuits and phenomics
Source: Commun Biol. 2024 Apr 18;7:477. doi: 10.1038/s42003-024-06187-5 (PMC11026520; doi:10.1038/s42003-024-06187-5)
Supplement: Supplementary file 2 — Supplementary Information [file 42003_2024_6187_MOESM2_ESM.pdf]

Supplementary Information

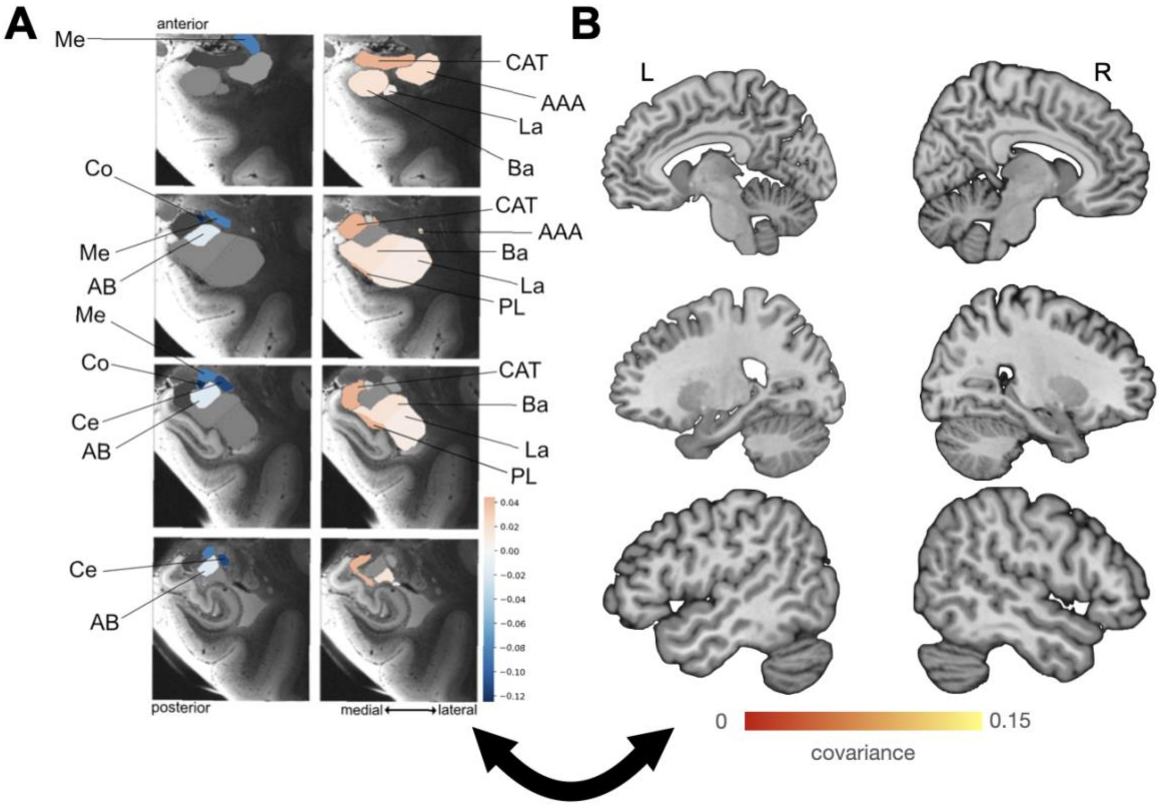

Supplementary Figure 1: Lateralization plasticity effects driven by the cortical, central, and medial nuclei. We determined how the ensuing subregion patterns lateralized in the 9 amygdala subregions and which cortical/subcortical regions are experiencing lateralization in the covariance of their structural plasticity with the lateralized amygdala subregions. The results also show the magnitude of the lateralized covariance in the regions experiencing lateralization among the 109 cortical/subcortical brain regions and among the 18 amygdala subregions. No color is shown for the brain regions that do not undergo robust lateralization effects . Shown here are the results of the bootstrap difference test as a form of variable selection in signature 2 which in turn conveys the lateralization in the amygdala subregion - brain region covariation. A) conveys the direction of lateralization of each of the 9 amygdala subregions in signature 3 [1]. The parameter weights of the subregions that robustly diverge between both hemispheres are depicted on 2 columns of 4 coronal slices of the amygdala parcellated into 9 subregions with each column portraying a different direction of lateralization occurring in each hemisphere. The subregions labelled with cold colours that are depicted in the left column exhibit the same direction of lateralization with varying magnitudes. Simultaneously, the subregions labelled with hot colours in the right column of coronal slices exhibit the opposite direction of lateralization to the subregions in the left column with each subregion having a distinct effect magnitude. While collective structural divergences exist in all the amygdala subregions, they are greatly pronounced in the cortical nucleus, central nucleus and medial nucleus. B) shows that no salient regions among the 109 cortical and subcortical regions were found to undergo hemispherically-biased covariation with the hemispherically-biased covariation of gray matter volume in the amygdala subregions. The absence of hemispherically-biased covariation parameter weights in the brain regions due to the lack of statistical robustness of the weights, indicates that the lateralization in the amygdala subregions occurs mainly due to the intra-amygdala interactions of the subregions with each other [2].

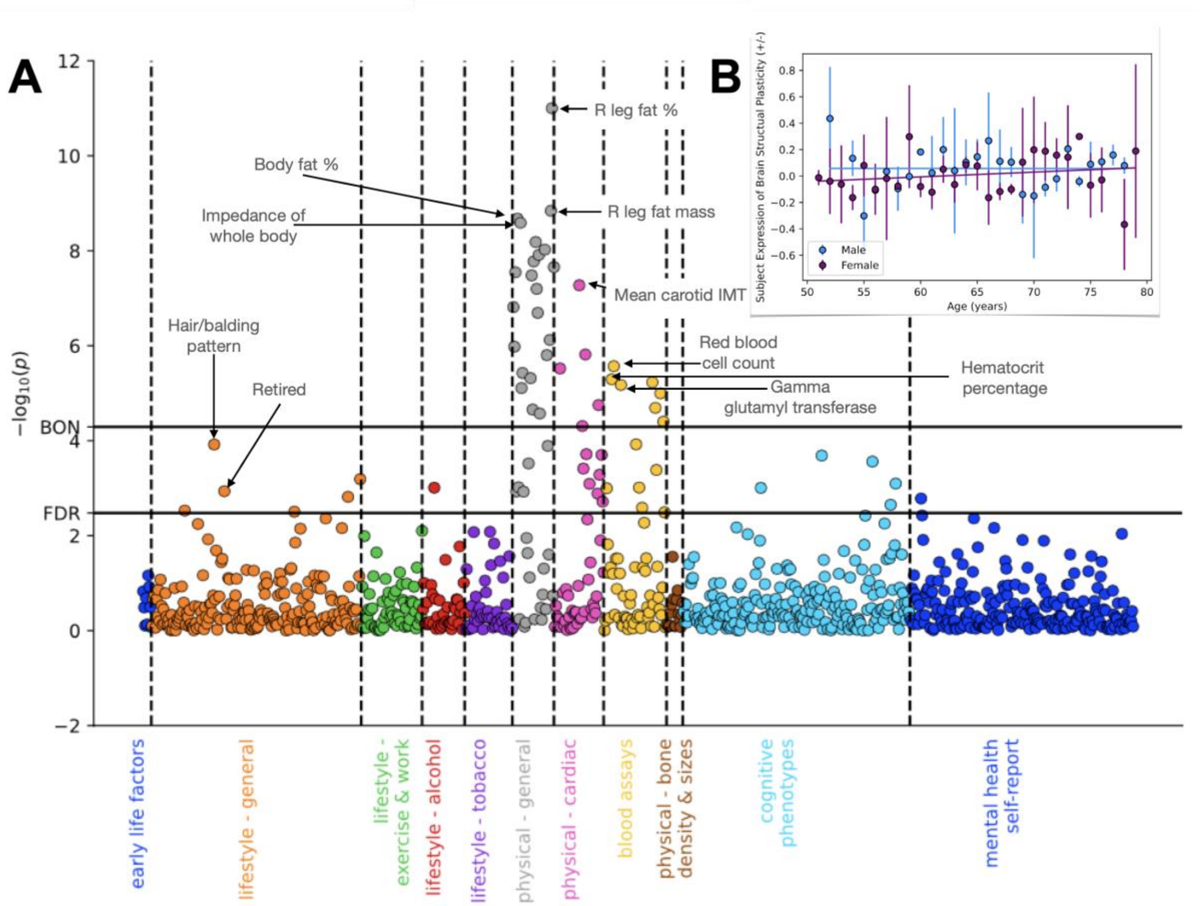

Supplementary Figure 2. Bonferroni threshold passed by body constitution, liver health marker (glutamyl transferase), and blood work indicator phenotypes linked to dominant AM-brain. plasticity pattern. A) Manhattan plot shows phenotype associations with individual (sub)cortical regions expressions in the first plasticity pattern (cf. Figure 1) in the UK Biobank population which charts 977 lifestyle indicators related variables divided across 11 domains. For each phenotype, the plasticity behaviour-links are shown as p-values (log. scale). Horizontal lines indicate the significance thresholds at Bonferroni correction (0.05/977) and at FDR correction for phenotypes. 65 phenotypes exceeded the FDR threshold and 34 exceeded the Bonferroni threshold in the first pattern. These significant phenotypes do not endorse or imply causality, but rather afford a valuable lens through which the amygdala-brain covariations can be contextualized. B) shows the median expression of the covariation pattern in the cortical and subcortical regions across age and sex of the first pattern which classify the differences in sex and age pattern strength. Error bars illustrate the lower 5th percentile and upper 95th percentile thresholds obtained by bootstrapping the median of the population, and two lines of best fit to the data are shown: the purple line corresponds to the female data while the blue line corresponds to the male data. The phenome-wide analysis showed significant associations with phenotypes related to body constitution phenotypes under the physical-general domain, liver health marker (glutamyl transferase) and blood work indicator phenotypes under the blood-assays domain which in turn helps translate the nature of the association that the primary analysis (cf. Figure 1) revealed between the central nucleus/anterior amygdaloid area and the inferior parietal lobule.

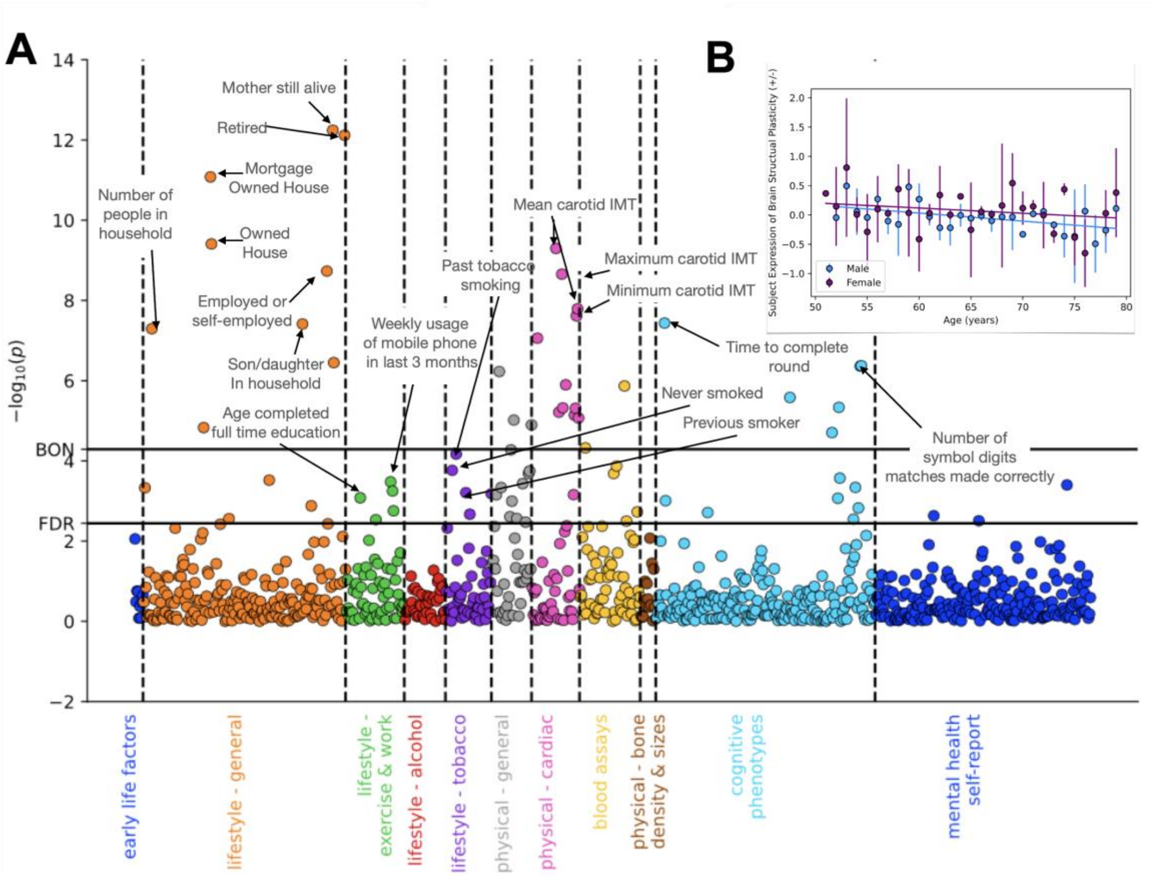

Supplementary Figure 3. Bonferroni threshold passed by household size, educational attainment, phone use frequency, smoking habits and cardiovascular risk factor phenotypes. A) Manhattan plot shows phenotype associations with individual (sub)cortical regions expressions in the second plasticity pattern (cf. Figure 2) in the UK Biobank population which charts 977 lifestyle indicators related variables divided across 11 domains. For each phenotype, the plasticity behaviour-links are shown as p-values (log. scale). Horizontal lines indicate the significance thresholds at Bonferroni correction (0.05/977). and at FDR correction for phenotypes. 70 phenotypes exceeded the FDR threshold and 31 exceeded the Bonferroni threshold in the second pattern. These significant phenotypes do not endorse or imply causality, but rather afford a valuable lens through which the amygdala-brain covariations can be contextualized. B) shows the median expression of the covariation pattern in the cortical and subcortical regions across age and sex of the second pattern which classify the differences in sex and age pattern strength. Error bars illustrate the lower 5th percentile and upper 95th percentile thresholds obtained by bootstrapping the median of the population, and two lines of best fit to the data are shown: the purple line corresponds to the female data while the blue line corresponds to the male data. The phenotype analysis showed significant associations with phenotypes related to financial well-being, number of people living in the household and if the people in the household are sons/daughters, physical health of the mother while other significant phenotypes found in this analysis are related to carotid IMT and phenotypes which are observed in cognitive tests.

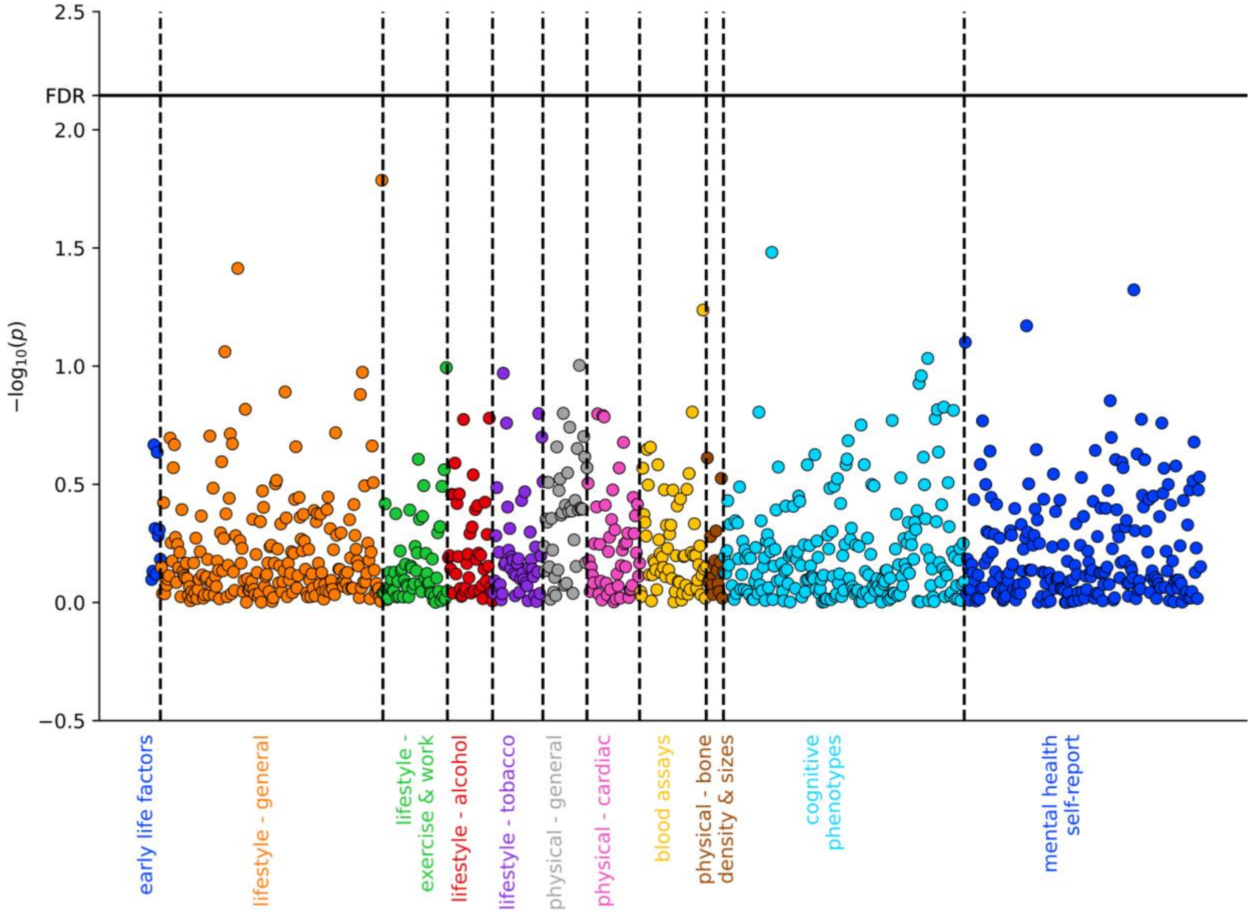

Supplementary Figure 4. No significant hits passed the FDR threshold in the fourth primary pattern. Manhattan plot shows phenotype associations with individual amygdala subregion expressions in the fourth plasticity pattern in the UK Biobank population which charts 977 lifestyle indicators related variables divided across 11 domains. For each phenotype, the plasticity behaviour-links are shown as p-values (log. scale). Horizontal lines indicate the significance thresholds at Bonferroni correction and at FDR correction for phenotypes (0.05/977). No phenotypes exceeded the FDR threshold or the Bonferroni threshold in the fourth pattern.

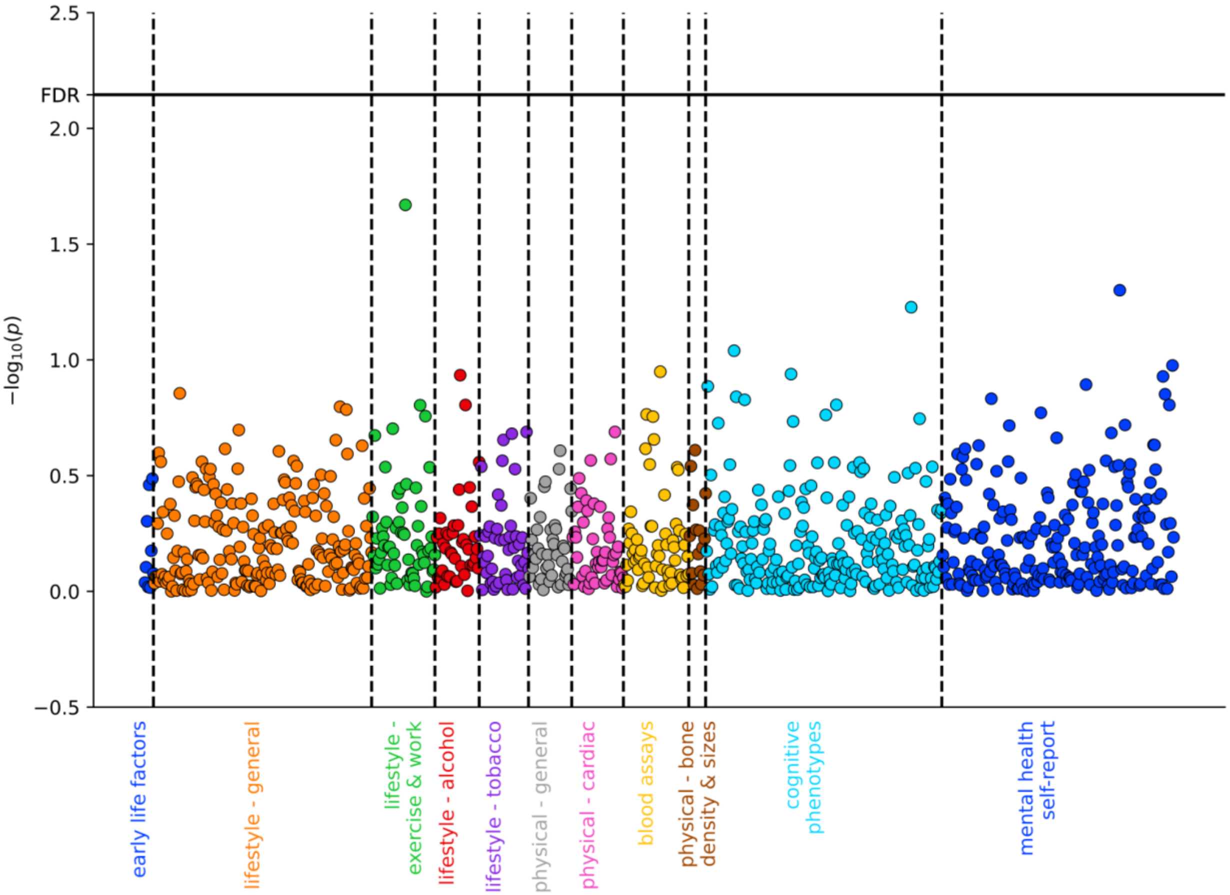

Supplementary Figure 5. No significant hits passed the FDR threshold in the fifth primary pattern. Manhattan plot shows phenotype associations with individual amygdala subregion expressions in the fifth longitudinal plasticity in the UK Biobank population which charts 977 lifestyle indicators related variables divided across 11 domains. For each phenotype, the plasticity behaviour-links are shown as p-values (log. scale). Horizontal lines indicate the significance thresholds at Bonferroni correction and at FDR correction for phenotypes (0.05/977). No phenotypes exceeded the FDR threshold or the Bonferroni threshold in the fifth pattern.

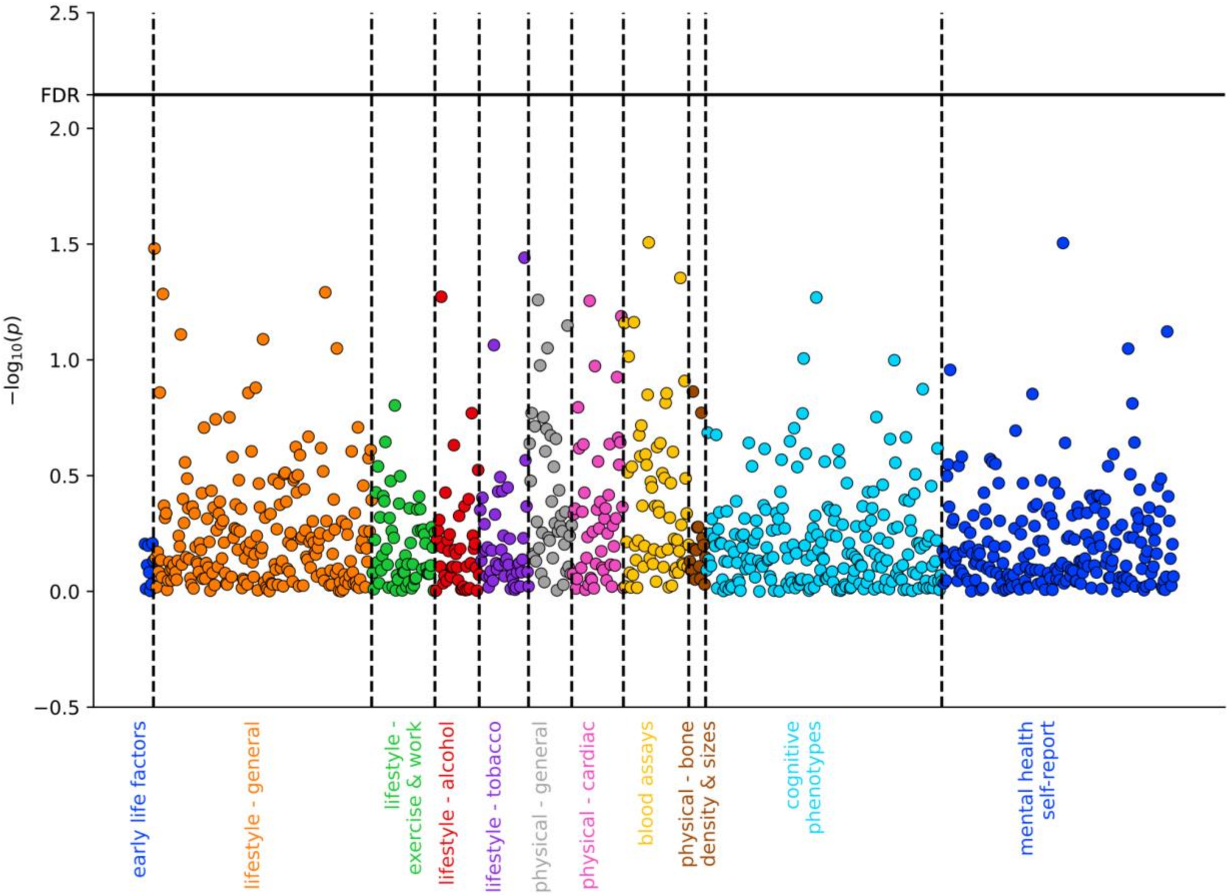

Supplementary Figure 6. No significant hits passed the FDR threshold in the sixth primary pattern. Manhattan plot shows phenotype associations with individual amygdala subregion expressions in the sixth plasticity pattern in the UK Biobank population which charts 977 lifestyle indicators related variables divided across 11 domains. For each phenotype, the plasticity behaviour-links are shown as p-values (log. scale). Horizontal lines indicate the significance thresholds at Bonferroni correction and at FDR correction for phenotypes (0.05/977). No phenotypes exceeded the FDR threshold or the Bonferroni threshold in the sixth pattern.

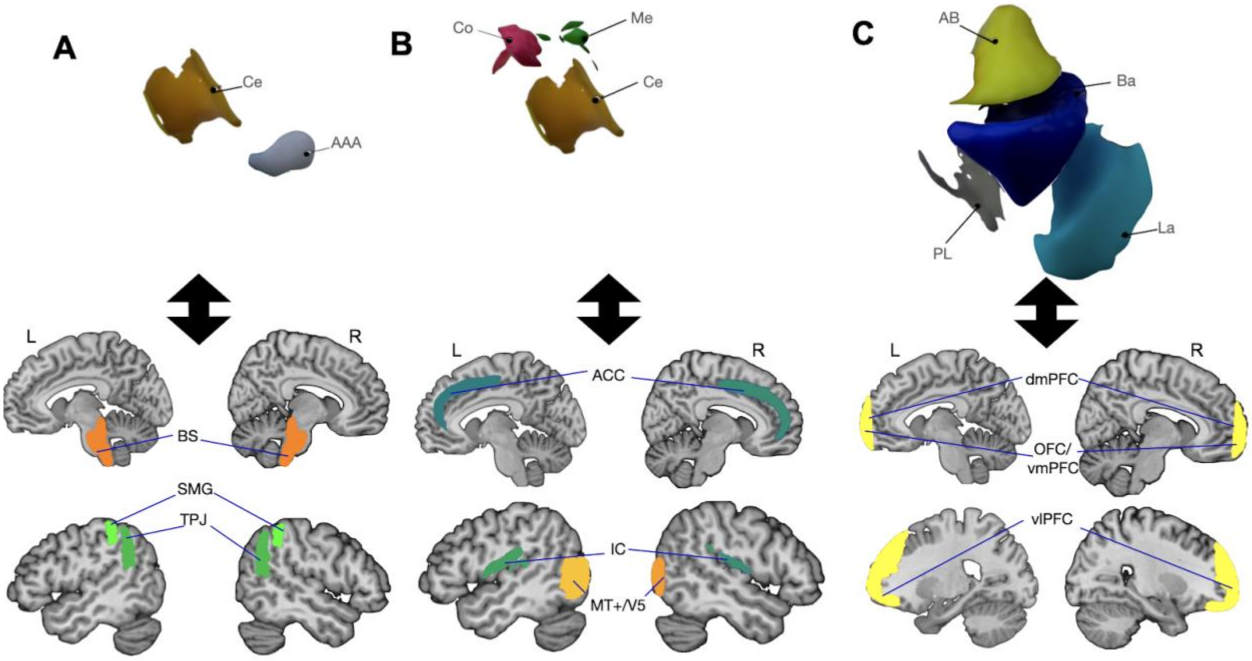

Supplementary Figure 7. Different types of circuits/relationships between the amygdala subregions [3] and the covarying brain networks A) Plasticity coupling in the first pattern of the central nucleus and the anterior amygdaloid area with right parietal cortex B) Plasticity coupling of the medial, cortical and central amygdala with brain regions related to alertness and visual conscious awareness C) Plasticity coupling of the laterobasal amygdala subregions with prefrontal cortex

Supplementary Table 1. Phenotype-wide association studies analysis table of significant hits (from largest to smallest hit association magnitude) above the Bonferroni correction threshold of the first pattern. The Magnitude - Phenotype column has the magnitude of the association between the phenotypes and the amygdala subregion-(sub)cortical region covariance which are shown in units as -log 10 of the p-values (left) and the name of the phenotype (right). The Category column shows under which of the 11 broad domains the phenotype lies.

Pattern 1			
**Magnitude - Phenotype**	**Category**	**Magnitude - Phenotype**	**Category**
11.0 - Leg fat percentage (right) (0.0)	PHYSICAL MEASURES - GENERAL	5.81 - Maximum carotid IMT (intima-medial thickness) at 240 degrees (2.0)	PHYSICAL MEASURES - CARDIAC & BLOOD VESSELS
8.84 - Leg fat mass (right) (0.0)	PHYSICAL MEASURES - GENERAL	5.8 - Hand grip strength (left) (0.0)	PHYSICAL MEASURES - GENERAL
8.67 - Body fat percentage (0.0)	PHYSICAL MEASURES - GENERAL	5.57 - Red blood cell (erythrocyte) count (0.0)	BLOOD ASSAYS
8.59 - Impedance of whole body (0.0)	PHYSICAL MEASURES - GENERAL	5.52 - Minimum carotid IMT (intima-medial thickness) at 240 degrees (2.0)	PHYSICAL MEASURES - CARDIAC & BLOOD VESSELS
8.18 - Impedance of arm (right) (0.0)	PHYSICAL MEASURES - GENERAL	5.43 - Impedance of leg (right) (0.0)	PHYSICAL MEASURES - GENERAL
8.02 - Arm fat percentage (left) (0.0)	PHYSICAL MEASURES - GENERAL	5.32 - Height (2.0)	PHYSICAL MEASURES - GENERAL
7.91 - Arm fat-free mass (left) (0.0)	PHYSICAL MEASURES - GENERAL	5.29 - Haematocrit percentage (0.0)	BLOOD ASSAYS
7.77 - Arm predicted mass (left) (0.0)	PHYSICAL MEASURES - GENERAL	5.23 - Gamma glutamyltransferase (0.0)	BLOOD ASSAYS
7.66 - Impedance of arm (left) (0.0)	PHYSICAL MEASURES - GENERAL	5.17 - Haemoglobin concentration (0.0)	BLOOD ASSAYS
7.55 - Whole body fat-free mass (0.0)	PHYSICAL MEASURES - GENERAL	5.11 - Sitting height (0.0)	PHYSICAL MEASURES - GENERAL
7.48 - Arm fat percentage (right) (0.0)	PHYSICAL MEASURES - GENERAL	5.0 - Urate (0.0)	BLOOD ASSAYS
7.27 - Mean carotid IMT (intima-medial thickness) at 240 degrees (2.0)	PHYSICAL MEASURES - CARDIAC & BLOOD VESSELS	4.75 - Mean carotid IMT (intima-medial thickness) at 120 degrees (2.0)	PHYSICAL MEASURES - CARDIAC & BLOOD VESSELS
7.2 - Arm fat-free mass (right) (0.0)	PHYSICAL MEASURES - GENERAL	4.69 - Creatinine (0.0)	BLOOD ASSAYS
6.81 - Leg fat-free mass (right) (0.0)	PHYSICAL MEASURES - GENERAL	4.65 - Impedance of leg (left) (0.0)	PHYSICAL MEASURES - GENERAL
6.69 - Hand grip strength (right) (0.0)	PHYSICAL MEASURES - GENERAL	4.57 - Trunk fat percentage (0.0)	PHYSICAL MEASURES - GENERAL
6.12 - Standing height (0.0)	PHYSICAL MEASURES - GENERAL	4.4 - Testosterone (0.0)	BLOOD ASSAYS
5.98 - Whole body water mass (2.0)	PHYSICAL MEASURES - GENERAL	4.31 - Systolic blood pressure	PHYSICAL MEASURES - CARDIAC & BLOOD VESSELS

Supplementary Table 2. Phenotype-wide association studies analysis table of significant hits (from largest to smallest hit association magnitude) above the Bonferroni correction threshold of the second pattern. The Magnitude - Phenotype column has the magnitude of the association between the phenotype and the amygdala subregion-(sub)cortical region covariance which are shown in units as -log 10 of the p-values (left) and the name of the phenotype (right). The Category column shows under which of the 11 broad domains the phenotype lies.

Pattern 2			
**Magnitude - Phenotype**	**Category**	**Magnitude - Phenotype**	**Category**
12.24 - Mother still alive (0.0)	LIFESTYLE AND ENVIRONMENT - GENERAL	6.23 - Impedance of whole body (0.0)	PHYSICAL MEASURES - GENERAL
12.12 - Current employment status (0.0)	LIFESTYLE AND ENVIRONMENT - GENERAL	5.9 - Mean carotid IMT (intima-medial thickness) at 210 degrees (2.0)	PHYSICAL MEASURES - CARDIAC & BLOOD VESSELS
11.08 - Own or rent accommodation lived in (0.0)	LIFESTYLE AND ENVIRONMENT - GENERAL	5.87 - Glycated haemoglobin (HbA1c) (0.0)	BLOOD ASSAYS
9.41 - Own or rent accommodation lived in (0.0)	LIFESTYLE AND ENVIRONMENT - GENERAL	5.58 - Interval between previous point and current one in alphanumeric path (trail #2) (2.0)	COGNITIVE PHENOTYPES
9.3 - Mean carotid IMT (intima-medial thickness) at 240 degrees (2.0)	PHYSICAL MEASURES - CARDIAC & BLOOD VESSELS	5.33 - Duration to complete alphanumeric path (trail #2) (2.0)	COGNITIVE PHENOTYPES
8.73 - Current employment status (0.0)	LIFESTYLE AND ENVIRONMENT - GENERAL	5.32 - Maximum carotid IMT (intima-medial thickness) at 210 degrees (2.0)	PHYSICAL MEASURES - CARDIAC & BLOOD VESSELS
8.65 - Maximum carotid IMT (intima-medial thickness) at 240 degrees (2.0)	PHYSICAL MEASURES - CARDIAC & BLOOD VESSELS	5.31 - Maximum carotid IMT (intima-medial thickness) at 120 degrees (2.0)	PHYSICAL MEASURES - CARDIAC & BLOOD VESSELS
7.79 - Mean carotid IMT (intima-medial thickness) at 150 degrees (2.0)	PHYSICAL MEASURES - CARDIAC & BLOOD VESSELS	5.21 - Systolic blood pressure	PHYSICAL MEASURES - CARDIAC & BLOOD VESSELS
7.62 - Minimum carotid IMT (intima-medial thickness) at 150 degrees (2.0)	PHYSICAL MEASURES - CARDIAC & BLOOD VESSELS	5.14 - Mean carotid IMT (intima-medial thickness) at 120 degrees (2.0)	PHYSICAL MEASURES - CARDIAC & BLOOD VESSELS
7.44 - Time to complete round (0.1)	COGNITIVE PHENOTYPES	5.07 - Maximum carotid IMT (intima-medial thickness) at 150 degrees (2.0)	PHYSICAL MEASURES - CARDIAC & BLOOD VESSELS
7.41 - How are people in household related to participant (0.0)	LIFESTYLE AND ENVIRONMENT - GENERAL	5.02 - Impedance of arm (right) (0.0)	PHYSICAL MEASURES - GENERAL
7.3 - Number in household (0.0)	LIFESTYLE AND ENVIRONMENT - GENERAL	4.9 - Impedance of arm (left) (0.0)	PHYSICAL MEASURES - GENERAL
7.06 - Minimum carotid IMT (intima-medial thickness) at 240 degrees (2.0)	PHYSICAL MEASURES - CARDIAC & BLOOD VESSELS	4.83 - Hair/balding pattern (0.0)	LIFESTYLE AND ENVIRONMENT - GENERAL
6.45 - Father still alive (0.0)	LIFESTYLE AND ENVIRONMENT - GENERAL	4.7 - FI7 : synonym (0.0)	COGNITIVE PHENOTYPES
6.37 - Number of symbol digit matches made correctly (2.0)	COGNITIVE PHENOTYPES	4.32 - IGF-1 (0.0)	BLOOD ASSAYS
6.36 - Number of symbol digit matches attempted (2.0)	COGNITIVE PHENOTYPES		

Supplementary Table 3. Phenotype-wide association studies analysis table of significant hits (from largest to smallest hit association magnitude) above the Bonferroni correction threshold of the third pattern. The Magnitude - Phenotype column has the magnitude of the association between the phenotype and the amygdala subregion-(sub)cortical region covariance which are shown in units as -log 10 of the p-values (left) and the name of the phenotype (right). The Category column shows under which of the 11 broad domains the phenotype lies.

Pattern 3			
**Magnitude - Phenotype**	**Category**	**Magnitude - Phenotype**	**Category**
69.48 - Height (2.0)	PHYSICAL MEASURES - GENERAL	9.85 - Impedance of leg (left) (0.0)	PHYSICAL MEASURES - GENERAL
65.54 - Standing height (0.0)	PHYSICAL MEASURES - GENERAL	9.66 - Total bilirubin (0.0)	BLOOD ASSAYS
60.27 - Whole body water mass (2.0)	PHYSICAL MEASURES - GENERAL	9.59 - Drive faster than motorway speed limit (0.0)	LIFESTYLE AND ENVIRONMENT - EXERCISE AND WORK
57.58 - Leg fat-free mass (right) (0.0)	PHYSICAL MEASURES - GENERAL	9.24 - HDL cholesterol (0.0)	BLOOD ASSAYS
57.06 - Hand grip strength (right) (0.0)	PHYSICAL MEASURES - GENERAL	8.86 - Platelet crit (0.0)	BLOOD ASSAYS
56.19 - Whole body fat-free mass (0.0)	PHYSICAL MEASURES - GENERAL	8.59 - Average total household income before tax (0.0)	LIFESTYLE AND ENVIRONMENT - GENERAL
55.94 - Leg fat percentage (right) (0.0)	PHYSICAL MEASURES - GENERAL	8.47 - QRS duration (2.0)	PHYSICAL MEASURES - CARDIAC & BLOOD VESSELS
55.07 - Sitting height (0.0)	PHYSICAL MEASURES - GENERAL	8.37 - Number of puzzles correctly solved (2.0)	COGNITIVE PHENOTYPES
53.6 - Hand grip strength (left) (0.0)	PHYSICAL MEASURES - GENERAL	8.36 - Own or rent accommodation lived in (0.0)	LIFESTYLE AND ENVIRONMENT - GENERAL
52.49 - Arm fat-free mass (left) (0.0)	PHYSICAL MEASURES - GENERAL	8.28 - Alanine aminotransferase (0.0)	BLOOD ASSAYS
52.05 - Arm fat-free mass (right) (0.0)	PHYSICAL MEASURES - GENERAL	8.2 - Hair/balding pattern (0.0)	LIFESTYLE AND ENVIRONMENT - GENERAL
50.5 - Arm predicted mass (left) (0.0)	PHYSICAL MEASURES - GENERAL	7.31 - Gamma glutamyltransferase (0.0)	BLOOD ASSAYS
42.49 - Forced vital capacity (FVC) (0.0)	PHYSICAL MEASURES - GENERAL	7.2 - Number in household (0.0)	LIFESTYLE AND ENVIRONMENT - GENERAL
40.53 - Body fat percentage (0.0)	PHYSICAL MEASURES - GENERAL	7.06 - IGF-1 (0.0)	BLOOD ASSAYS
40.44 - Arm fat percentage (right) (0.0)	PHYSICAL MEASURES - GENERAL	7.03 - Hair/balding pattern (0.0)	LIFESTYLE AND ENVIRONMENT - GENERAL
39.42 - Arm fat percentage (left) (0.0)	PHYSICAL MEASURES - GENERAL	6.72 - Illnesses of mother (0.0)	LIFESTYLE AND ENVIRONMENT - GENERAL
37.03 - Forced expiratory volume in 1-second (FEV1) (0.0)	PHYSICAL MEASURES - GENERAL	6.26 - Own or rent accommodation lived in (0.0)	LIFESTYLE AND ENVIRONMENT - GENERAL
33.42 - Testosterone (0.0)	BLOOD ASSAYS	6.14 - Time to complete round (0.1)	COGNITIVE PHENOTYPES
32.62 - Weight (pre-imaging) (2.0)	PHYSICAL MEASURES - GENERAL	6.09 - Whole body fat mass (0.0)	PHYSICAL MEASURES - GENERAL
31.9 - Leg fat mass (right) (0.0)	PHYSICAL MEASURES - GENERAL	6.07 - Hair/balding pattern (0.0)	LIFESTYLE AND ENVIRONMENT - GENERAL

30.49 - Impedance of arm (left) (0.0)	PHYSICAL MEASURES - GENERAL	6.06 - Weekly usage of mobile phone in last 3 months (0.0)	LIFESTYLE AND ENVIRONMENT - EXERCISE AND WORK
30.17 - Impedance of arm (right) (0.0)	PHYSICAL MEASURES - GENERAL	5.83 - Sensitivity / hurt feelings (0.0)	MENTAL HEALTH SELF-REPORT
25.8 - Weight (0.0)	PHYSICAL MEASURES - GENERAL	5.8 - Father still alive (0.0)	LIFESTYLE AND ENVIRONMENT - GENERAL
25.39 - Peak expiratory flow (PEF) (0.0)	PHYSICAL MEASURES - GENERAL	5.76 - Apolipoprotein A (0.0)	BLOOD ASSAYS
25.12 - Impedance of whole body (0.0)	PHYSICAL MEASURES - GENERAL	5.62 - Direct bilirubin (0.0)	BLOOD ASSAYS
24.85 - Haemoglobin concentration (0.0)	BLOOD ASSAYS	5.59 - Aspartate aminotransferase (0.0)	BLOOD ASSAYS
23.58 - Haematocrit percentage (0.0)	BLOOD ASSAYS	5.44 - Fluid intelligence score (0.0)	COGNITIVE PHENOTYPES
22.1 - Red blood cell (erythrocyte) count (0.0)	BLOOD ASSAYS	4.95 - Leisure/social activities (0.0)	LIFESTYLE AND ENVIRONMENT - GENERAL
19.02 - Waist circumference (0.0)	PHYSICAL MEASURES - GENERAL	4.77 - Lymphocyte count (0.0)	BLOOD ASSAYS
18.81 - Hair/balding pattern (0.0)	LIFESTYLE AND ENVIRONMENT - GENERAL	4.68 - Time spent driving (0.0)	LIFESTYLE AND ENVIRONMENT - EXERCISE AND WORK
16.52 - Trunk fat percentage (0.0)	PHYSICAL MEASURES - GENERAL	4.49 - Frequency of consuming six or more units of alcohol (0.0)	LIFESTYLE AND ENVIRONMENT - ALCOHOL
15.79 - Average weekly beer plus cider intake (0.0)	LIFESTYLE AND ENVIRONMENT - ALCOHOL	4.47 - Length of mobile phone use (0.0)	LIFESTYLE AND ENVIRONMENT - EXERCISE AND WORK
14.43 - Creatinine (0.0)	BLOOD ASSAYS	4.46 - Seen doctor (GP) for nerves	MENTAL HEALTH SELF-REPORT
13.6 - Urate (0.0)	BLOOD ASSAYS	4.4 - Qualifications (0.0)	LIFESTYLE AND ENVIRONMENT - GENERAL
12.11 - Impedance of leg (right) (0.0)	PHYSICAL MEASURES - GENERAL	4.4 - Time number displayed for (2.0)	COGNITIVE PHENOTYPES
11.71 - SHBG (0.0)	BLOOD ASSAYS	4.39 - Platelet count (0.0)	BLOOD ASSAYS
11.54 - Forced expiratory volume in 1-second (FEV1)	COGNITIVE PHENOTYPES	4.39 - Time to complete round (0.0)	COGNITIVE PHENOTYPES
10.9 - Current employment status (0.0)	LIFESTYLE AND ENVIRONMENT - GENERAL	4.3 - Number of incorrect matches in round (0.1)	COGNITIVE PHENOTYPES
10.59 - Sleeplessness / insomnia (0.0)	LIFESTYLE AND ENVIRONMENT - EXERCISE AND WORK		
10.03 - Current employment status (0.0)	LIFESTYLE AND ENVIRONMENT - GENERAL		

Supplementary References

1 Saygin, Z. M. *et al.* High-resolution magnetic resonance imaging reveals nuclei of the human amygdala: manual segmentation to automatic atlas. *Neuroimage* **155**, 370-382 (2017). https://doi.org:10.1016/j.neuroimage.2017.04.046

2 Pitkanen, A., Savander, V. & LeDoux, J. E. Organization of intra-amygdaloid circuitries in the rat: an emerging framework for understanding functions of the amygdala. *Trends Neurosci* **20**, 517-523 (1997). https://doi.org:10.1016/s0166-2236(97)01125-9

3 Elvira, U. K. A., Seoane, S., Janssen, J. & Janssen, N. Contributions of human amygdala nuclei to resting-state networks. *PLoS One* **17**, e0278962 (2022). https://doi.org:10.1371/journal.pone.0278962
