# Supplementary material for: Longitudinal microstructural changes in 18 amygdala nuclei resonate with cortical circuits and phenomics
Source: Commun Biol. 2024 Apr 18;7:477. doi: 10.1038/s42003-024-06187-5 (PMC11026520; doi:10.1038/s42003-024-06187-5)
Supplement: Supplementary file 5 — Reporting Summary [file 42003_2024_6187_MOESM5_ESM.pdf]

nature portfolio

Corresponding author(s): Karam Ghanem
Last updated by author(s): Apr 3, 2024

Reporting Summary

Nature Portfolio wishes to improve the reproducibility of the work that we publish. This form provides structure for consistency and transparency in reporting. For further information on Nature Portfolio policies, see our Editorial Policies and the Editorial Policy Checklist.

Statistics

For all statistical analyses, confirm that the following items are present in the figure legend, table legend, main text, or Methods section.

n/a	Confirmed	
☐	☒	The exact sample size (*n*) for each experimental group/condition, given as a discrete number and unit of measurement
☐	☒	A statement on whether measurements were taken from distinct samples or whether the same sample was measured repeatedly
☐	☒	The statistical test(s) used AND whether they are one- or two-sided<br>*Only common tests should be described solely by name; describe more complex techniques in the Methods section.*
☐	☒	A description of all covariates tested
☐	☒	A description of any assumptions or corrections, such as tests of normality and adjustment for multiple comparisons
☐	☒	A full description of the statistical parameters including central tendency (e.g. means) or other basic estimates (e.g. regression coefficient) AND variation (e.g. standard deviation) or associated estimates of uncertainty (e.g. confidence intervals)
☐	☒	For null hypothesis testing, the test statistic (e.g. F, t, r) with confidence intervals, effect sizes, degrees of freedom and P value noted<br>*Give P values as exact values whenever suitable.*
☒	☐	For Bayesian analysis, information on the choice of priors and Markov chain Monte Carlo settings
☒	☐	For hierarchical and complex designs, identification of the appropriate level for tests and full reporting of outcomes
☐	☒	Estimates of effect sizes (e.g. Cohen's d, Pearson's r), indicating how they were calculated

Our web collection on statistics for biologists contains articles on many of the points above.

Software and code

Policy information about availability of computer code

Data collection	Data are provided as freely available resource available to all scientists. No software was used for data collection.
Data analysis	Software used for data analysis:<br>FMRIB UKBioBank Normalisation, Parsing And Cleaning Kit (FUNPACK; version 2.5.0)<br>PHEnome Scan ANalysis Tool (PHESANT; https://github.com/MRCIEU/PHESANT)<br>sklearn (version: 0.24.2)<br>nilearn (version 0.9.0)<br>nibabel (version 3.2.2)<br>numpy (version: 1.20.3)<br>pandas (version 1.3.4)<br>seaborn (version: 0.11.2)<br>matplotlib (version: 3.4.3)<br>python (version 3.9.7)

For manuscripts utilizing custom algorithms or software that are central to the research but not yet described in published literature, software must be made available to editors and reviewers. We strongly encourage code deposition in a community repository (e.g. GitHub). See the Nature Portfolio guidelines for submitting code & software for further information.

Data

Policy information about availability of data

All manuscripts must include a data availability statement. This statement should provide the following information, where applicable:

- Accession codes, unique identifiers, or web links for publicly available datasets
- A description of any restrictions on data availability
- For clinical datasets or third party data, please ensure that the statement adheres to our policy

All used data are available to other investigators online (ukbiobank.ac.uk). https://fsl.fmrib.ox.ac.uk/fsl/fslwiki/Atlases.

Research involving human participants, their data, or biological material

Policy information about studies with human participants or human data. See also policy information about sex, gender (identity/presentation), and sexual orientation and race, ethnicity and racism.

Reporting on sex and gender	We identified the full set of ~40,000 UK Biobank participants with structural MRI images. Among these participants, 48% were men and were 52% women. We then only considered participants with structural MRI Images taken at two time points within the mentioned set. Hence, we only accounted for 1,414 healthy participants with longitudinal brain scanning from two different time points out of the ~40,000 participants and excluded the rest. The final set of participants consisted of 1,414 participants with 50.4% men and 49.6% women. Sex was self-reported. Sex was not considered when constructing structural plasticity patterns (deconfounding and z-scoring were done in a sex-independent manner). Sex effects were assessed after structrual plasticity patterns were derived to elucidate how structural plasticity differs between sexes (Panel B in Figure 6)
Reporting on race, ethnicity, or other socially relevant groupings	We did not report on race, ethnicity, or other socially relevant groupings. The omission of these variables was guided by the specific scientific questions at hand, which focused on neuroanatomical and behavioral changes without hypothesized links to such sociodemographic factors.
Population characteristics	40,000 UK biobank participants who are close to be representatively of the UK population, 47.5% men and 52.5% women, aged 40-69 years when recruited (mean age 54.9, standard deviation [SD] 7.5 years. The final set of participants consisted of 1,414 participants from the original 40,000 UK biobank participants with 50.4% men and 49.6% women who had two structural MRI Images taken at two time points with the the second visit ocurring ~2.3 years after each participant's first visit (min 24 months, max 36 months, [SD] 5 months). [The mean age at the first visit : 62.63 and the standard deviation at the first visit: 7.14. The mean age at the second visit : 64.89 and the standard deviation at the second visit: 7.16]
Recruitment	Recruitment was done as part of the UKbiobank initiative by flyers and other means common in epidemiological research. For details on representativeness see Frey et al., 2017.
Ethics oversight	UK Biobank participants gave written, informed consent for the study, which was approved by the Research Ethics Committee under application 11/NW/0382.

Note that full information on the approval of the study protocol must also be provided in the manuscript.

Field-specific reporting

Please select the one below that is the best fit for your research. If you are not sure, read the appropriate sections before making your selection.

☒ Life sciences ☐ Behavioural & social sciences ☐ Ecological, evolutionary & environmental sciences

For a reference copy of the document with all sections, see nature.com/documents/nr-reporting-summary-flat.pdf

Life sciences study design

All studies must disclose on these points even when the disclosure is negative.

Sample size	We used subject data from the ~40,000 UK Biobank release. We then only considered participants with structural MRI Images taken at two time points within the mentioned set which happens to be 1,414 healthy participants and excluded the rest of the participants. No power calculation was needed in advance.
Data exclusions	We first identified the full set of ~40,000 participants who had also been imaged by UK Biobank. We then only considered participants with structural MRI Images at two time points within the mentioned set. Hence, we ended up with 1,414 healthy participants with longitudinal brain scanning from two different time points out of the ~40,000 participants and excluded the rest of the participants.
Replication	All bootstrap replications were successful. As such, we have performed rigorous bootstrap resampling schemes: structural plasticity patterns were identified based on bootstrap-resampled subset of participants and assessed against structural plasticity patterns derived from the subset of 1,414 participants from the UKBB cohort
Randomization	UK Biobank is an observational prospective epidemiological study, and the analyses in our study use all available subjects that fulfill the

Randomization	criteria described above. Hence there is no equivalent process of randomization that comes into this analysis (this is not a controlled randomised study).
Blinding	For exactly the same reasons (this is not a controlled randomised study), there is no step equivalent to blinding involved.

Reporting for specific materials, systems and methods

We require information from authors about some types of materials, experimental systems and methods used in many studies. Here, indicate whether each material, system or method listed is relevant to your study. If you are not sure if a list item applies to your research, read the appropriate section before selecting a response.

Materials & experimental systems

n/a	Involved in the study
☒	☐ Antibodies
☒	☐ Eukaryotic cell lines
☒	☐ Palaeontology and archaeology
☒	☐ Animals and other organisms
☒	☐ Clinical data
☒	☐ Dual use research of concern
☒	☐ Plants

Methods

n/a	Involved in the study
☒	☐ ChIP-seq
☒	☐ Flow cytometry
☐	☒ MRI-based neuroimaging

Plants

Seed stocks	*Report on the source of all seed stocks or other plant material used. If applicable, state the seed stock centre and catalogue number. If plant specimens were collected from the field, describe the collection location, date and sampling procedures.*
Novel plant genotypes	*Describe the methods by which all novel plant genotypes were produced. This includes those generated by transgenic approaches, gene editing, chemical/radiation-based mutagenesis and hybridization. For transgenic lines, describe the transformation method, the number of independent lines analyzed and the generation upon which experiments were performed. For gene-edited lines, describe the editor used, the endogenous sequence targeted for editing, the targeting guide RNA sequence (if applicable) and how the editor was applied.*
Authentication	*Describe any authentication procedures for each seed stock used or novel genotype generated. Describe any experiments used to assess the effect of a mutation and, where applicable, how potential secondary effects (e.g. second site T-DNA insertions, mosiacism, off-target gene editing) were examined.*

Magnetic resonance imaging

Experimental design

Design type	Please see "Methods" for full details. Our analyses include data from Structural MRI (T1), and diffusion MRI.
Design specifications	MRI data processing (to generate imaging-derived phenotypes) was done previously and is fully described in references (Miller et al. 2016 and Alfaro-Almagro et al. 2018.).
Behavioral performance measures	Behavioral performance in the MRI scanner was not used in this study.

Acquisition

Imaging type(s)	Please see "Methods" for full details. Our analyses include data from Structural MRI (T1), and diffusion MRI
Field strength	3T
Sequence & imaging parameters	MRI data acquisition for the structural and functional modalities covers several pages of full detail, which is fully provided previously in references (Miller et al. 2016 and Alfaro-Almagro et al. 2018.).
Area of acquisition	Siemens' auto-align was used to include the full brain in the imaged field-of-view; this was checked (and corrected if necessary) by the radiographer.

Diffusion MRI ☐ Used ☒ Not used

Preprocessing

Preprocessing software	The processing pipeline used for the initial data release was primarily based on tools from FSL (the FMRIB Software Library) and is fully described in references (Miller et al. 2016 and Alfaro-Almagro et al. 2018.). Preprocessing included gradient distortion correction (GDC), field of view reduction using the Brain Extraction Tool(Smith et al., 2002), and FLIRT(Jenkinson et

	al., 2002; Jenkinson and Smith et al., 2001), and nonlinear registration to MNI152 standard space at 1-mm resolution using FNIRT (Mhuircheartaigh et al., 2010).
Normalization	Each subject's brain image is adjusted to align with the standard brain template using linear transformations (translations, rotations, scaling, and shearing). Further, more complex adjustments are made to match the subject's brain to the template using non-linear warping. This step accounts for the significant anatomical variability between individuals. Full description in references (Miller et al. 2016 and Alfaro-Almagro et al. 2018.)
Normalization template	MNI152 (linear alignment and non-linear warping) See above (covered previously in full detail in Miller et al. 2016 and Alfaro-Almagro et al. 2018).
Noise and artifact removal	Structured artifacts are removed by ICA + FIX processing. The data was adjusted for parameters that might otherwise induce apparent relationships based on potentially non-interesting factors (age, sex, head size, head motion). Full description in references (Miller et al. 2016 and Alfaro-Almagro et al. 2018.)
Volume censoring	No volume censoring. See above (covered previously in full detail in Miller et al. 2016 and Alfaro-Almagro et al. 2018).

Statistical modeling & inference

Model type and settings	n/a
Effect(s) tested	n/a

Specify type of analysis: ☐ Whole brain ☒ ROI-based ☐ Both

Anatomical location(s)	A set of ROIs were generated based on Harvard-Oxford atlas (109 regions from T1-weighted structural MRI); Another set of ROIs were extracted using FreeSurfer subsegmentation (18 ROIs based on T1-weighted MRI)
Statistic type for inference (See Eklund et al. 2016)	Our analysis was conducted using partial least squares canonical analysis (PLSC) to evaluate a relationship between the brain region variable set and the set of amygdala subregions.
Correction	FDR and Bonferroni corrections were used for testing linear association strength.

Models & analysis

n/a	Involved in the study
☒	☐ Functional and/or effective connectivity
☒	☐ Graph analysis
☐	☒ Multivariate modeling or predictive analysis

Multivariate modeling and predictive analysis	We used partial least squares canonical analysis (PLSC) for diffusion-weighted and structural MRI data
